# Textile Chemical Sensors Based on Conductive Polymers for the Analysis of Sweat

**DOI:** 10.3390/polym13060894

**Published:** 2021-03-14

**Authors:** Isacco Gualandi, Marta Tessarolo, Federica Mariani, Luca Possanzini, Erika Scavetta, Beatrice Fraboni

**Affiliations:** 1 Dipartimento di Chimica Industriale ‘Toso Montanari’, Università di Bologna, Viale Risorgimento 4, 40136 Bologna, Italy; erika.scavetta2@unibo.it; 2 Dipartimento di Fisica e Astronomia, Università di Bologna, Viale Berti Pichat 6/2, 40127 Bologna, Italy; marta.tessarolo3@unibo.it (M.T.); luca.possanzini2@unibo.it (L.P.); beatrice.fraboni@unibo.it (B.F.)

**Keywords:** conductive polymers, wearable sensors, textile sensors, fiber electronics, sweat analysis, potentiometric sensors, organic electrochemical transistor

## Abstract

Wearable textile chemical sensors are promising devices due to the potential applications in medicine, sports activities and occupational safety and health. Reaching the maturity required for commercialization is a technology challenge that mainly involves material science because these sensors should be adapted to flexible and light-weight substrates to preserve the comfort of the wearer. Conductive polymers (CPs) are a fascinating solution to meet this demand, as they exhibit the mechanical properties of polymers, with an electrical conductivity typical of semiconductors. Moreover, their biocompatibility makes them promising candidates for effectively interfacing the human body. In particular, sweat analysis is very attractive to wearable technologies as perspiration is a naturally occurring process and sweat can be sampled non-invasively and continuously over time. This review discusses the role of CPs in the development of textile electrochemical sensors specifically designed for real-time sweat monitoring and the main challenges related to this topic.

## 1. Introduction

The development of wearable chemical sensors has been attracting great attention due to the huge potential applications in the fields of medicine, occupational safety, health and sports [1]. Monitoring the body status and surrounding environment together with real-time data elaboration allows for the identification of hazards in order to quickly take countermeasures against potential damage. The collected information can also be shared and evaluated by specialized operators, boosting the point-of-care testing and the home health care service.

**Figure 1:**
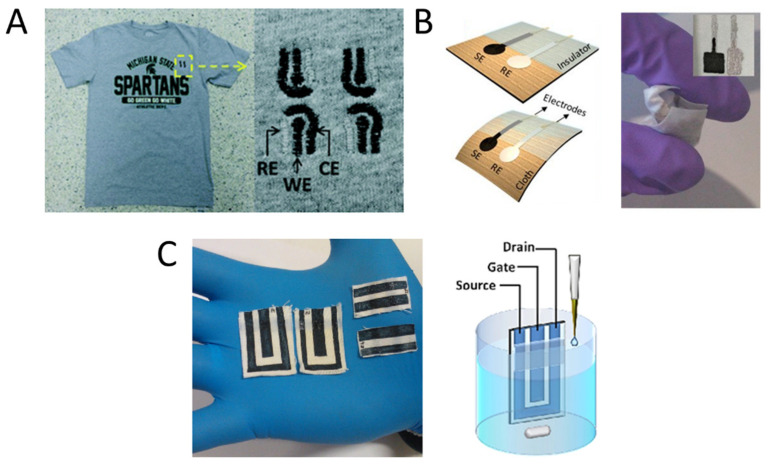
Examples of different kinds of electrochemical textile sensors. (**A**) Example of a textile amperometric sensor [17]. An amperometric sensor exploits the current associated to a redox reaction occurring at a working electrode (WE) as the analytical signal. The WE potential is usually fixed with respect to a reference electrode (RE) and both electrodes must be immersed in the same solution. The most rigorous measurements are performed in a three-electrode cell endowed with a counter electrode (CE). Reproduced from Ref. [16] with permission from the Royal Society of Chemistry. (**B**) Example of potentiometric sensors [16]. A potentiometric sensor is based on the measurement of the potential difference between the indicator electrode (SE) and a reference electrode (RE) that must be immersed in the solution under investigation. The Nernst equation describes the dependence of the potential on the logarithm of the analyte concentration. The signal stems from redox reactions involving the analyte or a membrane that selectively allows its adsorption or passage. (**C**) Example of an organic electrochemical transistor (OECT) printed on a cotton fabric [20]. OECTs are constituted of a channel made of a conductive polymer and a gate electrode. Both elements must be immersed in an electrolytic solution. When a voltage is applied to the gate electrode, electrochemical reactions take place at the channel that vary the charge concentration in the conductive polymer. Any substance that changes the current flow in the channel can be detected by these devices.

It should be noted that wearable electronics differ from mobile devices because of their aspect and functionality that should be designed to work while the device is worn on the body. A true wearable sensor should be worn internally, as implanted sensors, or externally to function, and thus it is conceptually linked to the wearer’s body [2]. Consequently, wearable devices must answer new constraints to be a winning technology, because besides being reliable when compared with the state-of-the-art conventional sensors, they should be designed to be as less invasive as possible, in view of reaching a wear-and-forget functionality. To satisfy these requirements, the literature proposes different design strategies that are usually based on embedding the sensing element in real-life objects, such as contact lenses [3,4], tattoos [5,6,7], garments [8,9], dental appliances [10] and medical dressings [11]. Of course, these gadgets should detect the target compounds in a specific biofluid in their proximity, and thus mouth guard sensors operate in saliva, textile sensors in sweat, and contact lenses in tears. Among different bio-fluids, human perspiration is the only one that can be collected continuously outside the body [12]. As sweat does not require the implantation of the sensing device, it is the only matrix that can guarantee noninvasive and safe measurements. Since it is collected on the skin surface, sweat sensors can be embedded into t-shirts, bands, underwear or cuffs. Although some optical devices have been fabricated [13,14], the electrochemical transduction is mainly employed due to the simplicity of the detection architecture wherein the chemical information is directly converted into an electrical signal at the electrode/electrolyte interface without the use of additional components such as lamps, monochromators and light detectors. Amperometric and potentiometric sensors are based on current and potential measurements, respectively, whose variation can be quantitatively correlated with the analyte concentration [15,16,17], and the amplification is performed by the read-out electronics. An organic electrochemical transistor (OECT) can perform the reading and amplification of these signals at the same time in the place of the measurement [18,19,20]. In addition to an increased signal quality, an amplified current or potential can be more easily collected by the read-out electronics that, consequently, can be simpler and smaller. Figure 1 describes examples of configurations of these sensors.

When chemical sensors are embedded in a noninvasive way in clothes, some properties of the textile should be preserved. The device should be low-weight and flexible to be comfortable, and, at the same time, it should permit the flows of heat, air, and humidity that usually occur in garments. In addition, it should absorb low-power and require a simple read-out electronics to be effectively portable. Conventional conductor materials fail to efficiently meet these requirements due to their rigidity and bulkiness [1,21].

The development of electrochemical textile sensors takes advantage of the recent progress in fiber and textile electronics [21] to produce conductive fabrics and yarns that will compose the device. A thin conductive film is usually deposited on commercial textiles by screen-printing [20,22], dip coating [23,24], spinning [25,26], electrochemical deposition [27], oxidative ink-jet printing [28], in-situ polymerization [29,30], physical vapor deposition [31] and magnetron sputtering [32]. The mainly employed conductive species are nanomaterials based on metals and carbon, and conductive polymers. On the other hand, conductive yarns can be prepared by electrospinning [33], wet spinning [34], melt spinning [35] and floating catalyst vapor deposition [36].

**Figure 2:**
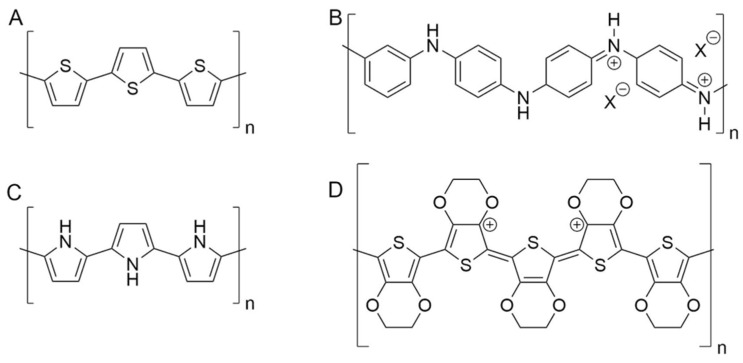
Chemical structure of the main conductive polymers (CPs) employed in textile sensors. (**A**) Polythiophene; (**B**) polyaniline; (**C**) polypyrrole; (**D**) poly (3,4-ethylendioxythiopene).

Conductive polymers (CPs) are fascinating materials for these applications because they are characterized by electrical conductivities lying in the range of semiconductors or even metals [29]. The carbon atoms constituting the polymer backbone are sp^2^ hybridized. Therefore, the p_z_ orbitals are available for the formation of a conjugated Π system that involves the polymer chain in its whole length. Since the charge carrier species, usually in the form of holes, are free to move along the chain, electricity can easily pass through these materials. CPs can be doped by the extraction of electrons through redox processes, with the formation of polaron and bipolaron states in the region between valence and conduction bands. Moreover, charge carriers can be stabilized by mixing the CPs with oppositely charged polyions. For these reasons, the electrical conductivity of CPs lies in the range of semiconductors [29]. At the same time, the polymeric structure is less rigid and more deformable than the crystal lattices of conventional conductors and semiconductors. In addition, CPs’ mechanical features depend on their chemical composition and can be varied either by adding specific compounds such as plasticizers [37,38] and by preparing polymeric blends [39,40]. In addition to the mechanical compatibility with skin and fibers, the most important CPs are also biocompatible [41,42,43] and can be placed in close contact to the skin [5]. CPs are usually processed by soft techniques that do not require harsh experimental conditions (such as high temperature or aggressive chemicals), which could degrade commercial textiles [20,23]. Last but not least, CPs exhibit peculiar properties that are widely exploited in sensing. The ability to electro-catalyze the oxidation of several compounds permits the conversion of a chemical signal in a current, and thus the production of amperometric sensors for the detection of these analytes [44,45]. At the same time, as CPs are both ionic and electronic conductors, they can easily convert these kinds of signals into each other [46]. This feature is exploited for recording bio-potentials, which have an ionic nature and must be converted in an electronic signal, which is the only one readable by read-out electronics. Furthermore, CPs can be used also in potentiometric sensors, since a thin layer of CP placed between the electrode and the ion-selective membrane increases the signal stability [45]. The main CPs used in fiber electronics belong to the classes of polypyrroles, polyanilines and polythiophenes (see Figure 2). Among polythiophenes, poly(3,4-ethylenedioxythiophene):polystyrene sulfonate (PEDOT:PSS) plays a key role due to the good electrical conductivity, reversible doping/dedoping process and high thermal and chemical stability.

CPs’ success is demonstrated by their large use in the fabrication of textile appliances such as batteries, solar cells, energy harvesting devices and sensors. Gadgets based on smart textiles have been developed for the physical sensing of bio-potentials (such as electrocardiogram, electromyogram, electroencephalogram) [47,48], pressure [49] and temperature [50]. In addition, CPs were employed for the fabrication of textile gas sensors based on chemiresistor transduction [51,52,53].

This review deals with the use of CPs in the fabrication of textile chemical sensors for sweat analysis and it is structured as follows. Firstly, we focus our attention on the fabrication of textiles modified with CPs and we briefly describe the scientific background concerning sweat analysis. Then, the textile chemical sensors based on CPs are classified considering the target analyte and discussed considering the role of these materials in the sensing mechanism. Finally, the main challenges in the development of textile sensors based on CPs are analyzed.

## 2. Fabrication of Conductive Textile Based on Conductive Polymers

The first step for producing textile sensors is the preparation of conductive filaments or textiles. As with other polymeric materials, CPs are usually mixed with specific compounds to tune the physical properties of the conductive layer. Therefore, the preparation techniques usually produce a conductive polymer composite (CPC) because these additives remain entrapped in the film. There are three main ways to produce a conductive textile based on CPs: (i) a thin layer of CPC can be deposited onto a commercial textile by coating/dyeing or printing a solution/suspension containing the polymer; (ii) a thin layer of CP can be directly polymerized onto the textile by in situ polymerization, vapor phase polymerization or electropolymerization; (iii) the CPC can be spun in the form of fiber by wet spinning, melt spinning or electrospinning alone or in combination with other polymers. Then, the conductive fibers are spun to produce yarns. It is worthy to note that coating, dyeing, printing, or in situ polymerization are useful to functionalize the surface of a yarn or a fabric, while spinning techniques allow to create fibers for the production of conductive yarns.

**Figure 3:**
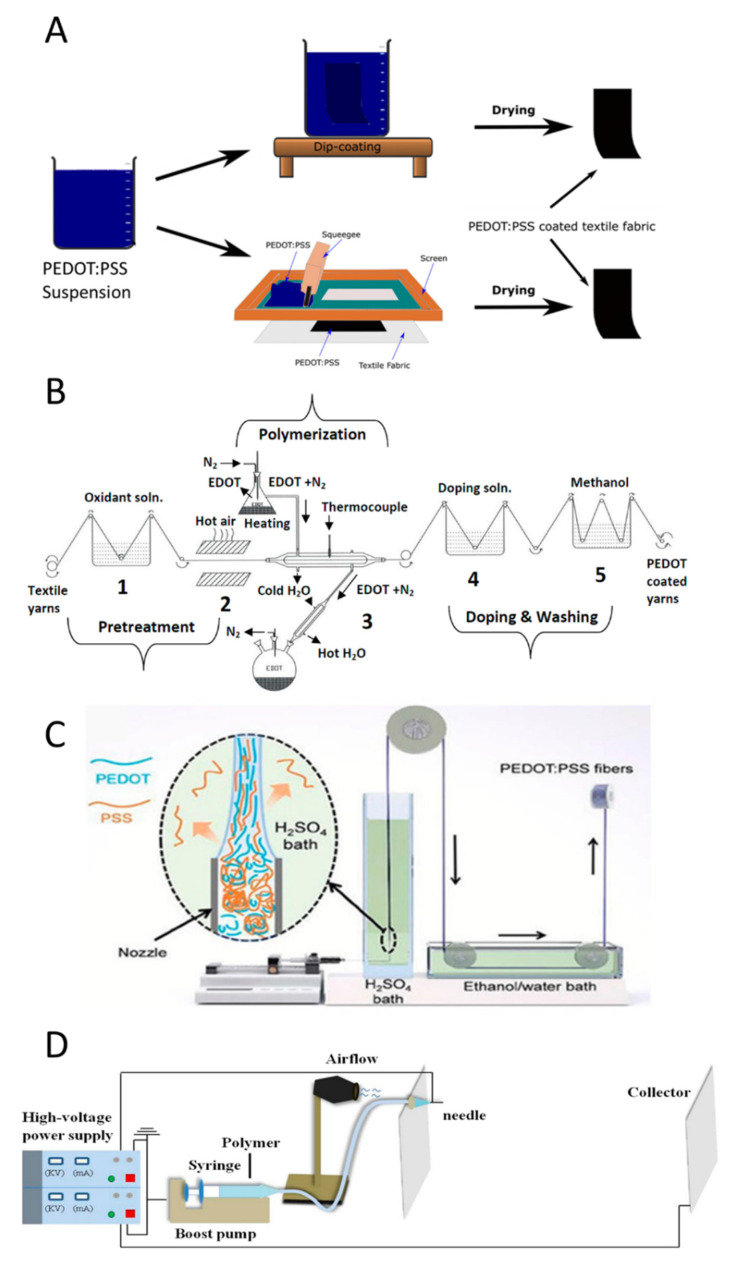
Fabrication process of conductive textile based on CPs. (**A**) Processes starting from a CPs suspension: dip-coating (top) and screen-printing (down) [56]. (**B**) Automatized plant for the chemical vapor deposition of poly(3,4-ethylenedioxythiophene):polystyrene sulfonate (PEDOT:PSS) on a yarn [57]. (**C**) Schematic illustration of a modified set-up used for the wet spinning of PEDOT:PSS fibers. Inset shows the schematic illustration of the alignment of PEDOT:PSS chains during the fiber formation and the outward diffusion of excess PSS to H_2_SO_4_ coagulation bath. Reproduced from Ref. [34] with permission from the Royal Society of Chemistry. (**D**) Schematic diagram of the electrospinning setup. Adapted from [33].

When the conductive film is prepared by deposition [23] or printing [20] (Figure 3A), a solution/suspension is placed on the textile and, after solvent evaporation, the CP precipitates on the textile. Several commercial products based on CPs are available on the market, but their composition should be varied to answer the peculiarity of the different deposition processes. For example, dip-coating and spin coating is needed for CP solutions with a low viscosity, because they have to wet the textile to ensure the continuity of the film [21]. On the other hand, screen-printing and stencils require very viscous inks to hinder the percolation outside the masked area with a consequent reduction of the pattern resolution [20]. Beyond the peculiarity associated to the deposition techniques, auxiliary components are added to impart specific features to the resulting CPC film. For example, secondary dopants enhance the conductivity by acting on the microstructure of the polymer [54], while plasticizers [55] increase the flexibility of the film and improve the resiliency of electrical features after deformation. In the coating/dyeing, the textile is immersed/dipped in the CP suspension/solution; consequently, the conductive film is deposited all over the surface. Conversely, the printing processes (such as screen-printing and ink-jet printing) allow one to deposit the CP film on a selected area of the textile. Both dyeing and printing processes are widely used in the textile industry, and thus offer the advantage of straightforward scaling-up.

The textile surface can also be coated by polymerization, starting from the monomeric units. The first step of a polymerization reaction is the monomer oxidation, to form radical cations. The following coupling reactions lead to the formation of the polymeric chain. During chemical vapor phase polymerization (Figure 3B), the monomer is in gas phase, while the oxidant is adsorbed on the textile [57,58,59]. Consequently, the polymerization takes place only on the fabric surface that will be covered by the CP thin film. The in-situ polymerization is performed by immersing the fabric in the solution containing the monomer and the oxidant. The reaction can be limited to the surface, when the impregnated textile is removed from the reactive solution and the oxidation is induced by a stimulus such as heating [30]. Electrochemical polymerization must be performed on a conductive textile soaked in a solution containing the monomer and an electrolyte [27]. The application of an anodic potential between the substrate and a reference electrode leads to the monomer oxidation and thus to the polymer formation.

Finally, wet spinning or electrospinning are employed to produce conductive fibers. In wet spinning (Figure 3C), the polymer is solubilized in a solvent, and the spinneret is submerged in a bath where the polymer is not soluble. When the solution is injected in the bath, the polymer precipitates in form of fibers by drying under tension. Zhang et al. have described a very interesting approach to wet spin a PEDOT commercial suspension using a strong acidic environment [34]. During the fiber formation, PSS is expelled from the conductive polymer, increasing the conductivity of the fiber. In melt spinning, CP and additives are heated to form a melt mixture suitable for the extrusion through the spinneret occurring into an air chamber, wherein cooling leads to the solidification into continuous fibers that are wound on spools [35,60]. Electrospinning takes advantage of electric forces to stretch the polymer droplets and make the fibers. CPs can be blended with other polymers to improve the mechanical features of the resulting CPC fibers [56].

## 3. Sweat Analysis

The composition of human perspiration has been investigated since the 19th century in order to identify a correlation with physiological states, diseases, or the use of drugs [61]. Although the literature describes promising results as far back as the 1940s and 1950s [12], today, sweat analysis is only routinely employed in cystic fibrosis diagnostics and for the detection of illicit drug use [61]. The interest in wearable technologies has stimulated the recent publication of some clinically oriented reviews [61,62] concerning sweat analysis. Here, we would like to provide a brief overview of the topic. The low and variable sweat rate, the sample evaporation and contamination, and the difficult sampling are technical constraints that have hindered the large use of sweat in clinical analysis [61]. Nevertheless, human perspiration is the only bio-fluid that can be sampled in real-time noninvasively, because the sensors can be placed on the skin (i.e., outside the body) in the proximity of eccrine sweat glands that secrete a very low but continuous amounts of sample.

Human perspiration is generated by eccrine sweat glands and some publications describe it as a blood filtrate, but sweating is a complex process that requires a thorough study of the partitioning process and the liquid transport to the skin surface. A human eccrine gland is composed of the secretory coil, the dermal duct, and the upper coiled duct, also named acrosyringium [63]. The secretory coil contains three primary cells: (i) the clear cells that are hypothesized to be the dominant source of sweat secretion; (ii) the dark ones, which are involved in membrane cellular transport and can also act as clear cells; (iii) myoepithelial cells acting as support. The epidermal duct is simpler and contains a bilayer of basal and luminal cells. In the acrosyringium, the duct slightly expands in diameter, until it emerges at the surface of the skin. As other metabolites (glucose, lactate, creatinine, urea, K^+^, Mg^2+^, Ca^2+^, NH_4_^+^, …) diffuse in human perspiration from the surrounding cells during the secretion, it could provide a picture of the chemical composition of the adjacent biological environment [12]. Since our bodies finely control the blood composition, while human perspiration composition also depends on secretion rate, sweat analysis cannot reach the reliability of a blood test, but it contains a lot of valuable information about our body state. Table 1 shows the main metabolites in sweat and their concentration.

## 4. Textile Chemical Sensors

### 4.1. pH

Proper pH regulation in the human body is essential to sustain a variety of physiological events and vital functions. Real-time and non-invasive monitoring of epidermal pH can give direct access to our body status, particularly concerning tissue integrity and wound healing progression, as well as sweat rate and dehydration [74]. The sweat pH of a healthy person lies in the range 4.5–6.5 and alkalinization up to pH 9 has been reported for people affected by cystic fibrosis [75]. Moreover, the pH of sweat has been studied as a biomarker to monitor sport activity intensity and related metabolic alkalosis [12,76], it has been correlated with blood glucose levels [77] and reflects the status of the acid mantle protecting our skin from diseases and dermatitis [78].

**Figure 4:**
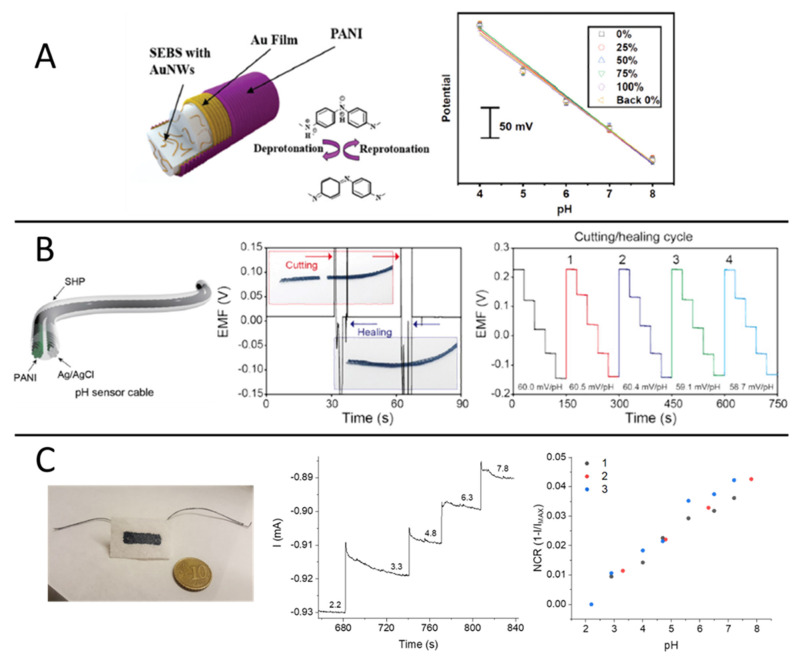
(**A**) Gold fiber-based wearable pH sensor. Structure of the Au nanowires (Au NWs)-containing styrene-ethylene/butylene-styrene (SEBS) fibers coated by Au film and electrochemically polymerised polyaniline (PANI) (left). Sensor response during stretchability tests (right) [81]. (**B**) Self-healable pH sensor cable. Structure of the pH sensor cable based on carbon fiber thread (CFT) electrodes coated with self-healing polymers (SHP) (left). Potentiometric responses of the pH sensor cable during multiple cutting and healing cycles (right) (reproduced from [82] with permission from Elsevier). (**C**) pH sensing bandage. Picture of the two-terminal wearable sensor based on electropolymerized dye-doped PEDOT on screen-printed PEDOT:PSS (left). Current vs. time response to pH variations in buffer solution (V = −200 mV) and repeatability of the normalized current response vs. pH (right) (reproduced from [27] with permission from Elsevier).

In laboratory practice, pH sensing is a routine procedure that benefits from one of the most robust electroanalytical tools, e.g., the glass membrane electrode. Unfortunately, bulkiness and brittle components evidently make this approach totally incompatible with wearable technologies. In contrast, solid-state potentiometric devices represent a viable strategy that has been introduced only in recent years for the design of textile pH sensors. The first works reporting on pH sensing textiles made use of an electrochemically deposited metal oxide film (IrO_2_) [79] as the pH-sensitive material for indicator electrode design. While IrO_x_ is a well-established and biocompatible potentiometric transducer in electrochemistry, later works targeting sweat analysis were exclusively devoted to the development of CP-based pH sensors due to their superior flexibility, low cost and inherent compatibility with textiles manufacturing processes. Among CPs, polyaniline (PANI) stands out as the most prominent example of non-redox doping caused by acid/base equilibria between deprotonated (e.g., base, dedoped) and protonated (e.g., salt, doped) forms and has been exploited for the design of textile potentiometric sensors. In particular, PANI electropolymerization has been carried out on wet-spun PEDOT:PSS fibers [80] and elastomeric gold fibers woven into a textile matrix [81] (Figure 4A), both showing good resiliency of the sensing performance during stretchability tests. In the first case, treatment with the organic solvent dimethyl sulfoxide (DMSO) increased the conductivity of the PEDOT:PSS fiber, thus facilitating PANI electrodeposition, and the sensor response covered the pH range 3.0–7.0 with Nernstian sensitivity (−56 ± 7 mV.pH^−1^). In the latter, a 60.6 mV pH^−1^ sensitivity was achieved in the pH range 4–8 with good specificity in the presence of NH_4_^+^, Ca^2+^, Mg^2+^ and Na^+^, while only a slight decrease in the woven sensor performance was reported during pH detection in artificial sweat.

Interestingly, a self-healable textile sensor was realized on PANI-coated carbon fiber threads (Figure 4B), which were eventually sewn into a sport headband for proof-of-concept pH monitoring during physical exercise [82]. The thread-based sensor exhibited Nernstian behavior (with a sensitivity of 58.28 mV·pH^−1^) within the pH range 3.89–10.09 and autonomous healing capability during four cutting/healing cycles, with no loss in the sensing performance during pH detection in sweat samples. Despite the evident advancements in sensor fabrication and material engineering, the production of stable reference electrode analogues on textile substrates remains a crucial bottleneck in the realization of potentiometric chemical sensors.

A different approach was recently proposed based on a PEDOT:PSS/dye-doped PEDOT film [27]. Thanks to the spontaneous electrochemical gating originated by the potentiometric transducer PEDOT:Bromothymol Blue (PEDOT:BTB), a pH-dependent modulation of the current flowing across the PEDOT:PSS film was obtained. Despite the referenceless, two-terminal device architecture, which is as simple as a chemoresistor, the robustness of this approach was demonstrated through the fabrication of a screen-printed pH sensor on a bioceramic fabric (Figure 4C). The textile pH sensor showed a normalized sensitivity of (7.5 ± 0.3) × 10^−3^ pH^−1^ in the range 2–7, with no penalization of the sensing performance if compared to the rigid analogous fabricated on a glass substrate. The same functionalization strategy based on PEDOT:PSS/PEDOT:BTB was also adopted to design a thread pH sensor [83]. The textile sensor was capable of detecting pH selectively during simultaneous recordings in combination with another thread-based sensor for Cl^−^ detection and will be discussed in the section entitled “Multi-sensing platforms”.

### 4.2. Ions

Ions such as Na^+^, Cl^−^, K^+^, NH_4_^+^ are essential components of human perspiration secreted by the endocrine glands. Despite the fact that sweat composition largely varies throughout the human body, the high correlation factor between different body regions allows one to predict with high precision the concentration of systematic electrolytes such as Na^+^ and Cl^−^ [65]. Besides, diet, heat adaptation rate, and genetic susceptibility may also affect ions’ concentration in different subjects.

In contrast, aging and gender do not affect the concentration of electrolytes in sweat [84]. On the other hand, a large amount of clinical and health-related information can be extracted from electrolytes’ analysis. Although Na^+^ and Cl^−^ concentrations in sweat have a limited correlation with the corresponding values in the blood, there is ample evidence that there is a correlation between Cl^−^ levels and medical conditions.

When an abnormally high chloride ion concentration is detected, it is possible to assess a non-invasive cystic fibrosis diagnosis or monitor hormonal change. Furthermore, thanks to the Na^+^ and Cl^−^ concentration values, hydration level [85] and hyponatremia can be identified, and, at the same time, the sweating rate can be indirectly measured [86]. In contrast, the concentration of potassium ions in sweat is directly related to the concentration of potassium ions in the blood, without the sweating rate having any effect on it. Furthermore, knowing the potassium concentration in sweat can provide important information about muscle activity [87] and a wide range of situations related to hyperkalemia or hypokalemia [88].

**Figure 5:**
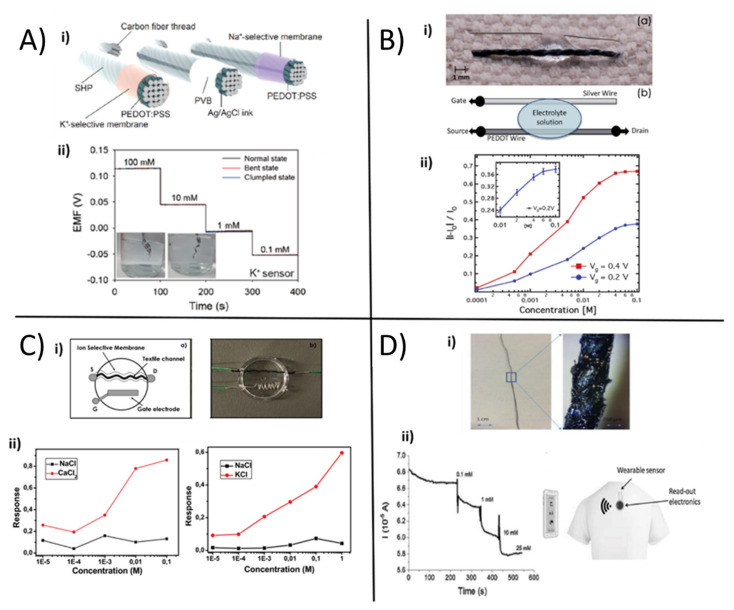
(**A**) i. Schematic illustration of self-healing ion-sensing and reference thread electrodes; ii. electrochemical characterization of sweat K^+^ sensor (adapted with permission from [91] Copyright (2021) American Chemical Society; (**B**) i. OECT based on a single cotton thread integrated on fabric. The bottom thread is the PEDOT wire channel while the upper wire is the Ag gate electrode; ii. plot of the normalized OECT response recorded at different gate voltages as a function of the salt concentration (reproduced from [92] with permission from the Royal Society of Chemistry). (**C**) i. textile ion selective OECT sketch with the ion selective membrane around textile channel and Ag gate electrode, ii. responses of textile -OECT as function of CaCl_2_, Na and KCl at gate voltage of 1V (adapted from [93] with permission from Elsevier); (**D**) i. optical microscopy images of a cotton thread modified with the PEDOT:PSS/Ag/AgCl NPs composite material; ii. textile SP/NP-based sensor response while Cl^−^ concentration was increased (reproduced from [94] with permission from Elsevier). PVB: polyvinyl butyral.

Following the footsteps of the pioneering works of Parrilla [89] and Guinovart [90] on textile sensors using the ion selective membrane (ISM) as a sensing element to realize a textile sensor for ions concentration in sweat, Yoon et al. [91] proposed high-sensitive wearable sensors endowed with self-healing properties based on carbon fibers, PEDOT:PSS, ISMs and Poly(1,4-cyclohexanedimethanol succinate-cocitrate) (PCSC). The wearable sweat-sensing device consisted of an indicator and a reference electrode sewn in a textile substrate with Bluetooth transmission technology (Figure 5A i). After coating carbon nanotubes (CNTs) with the conductive polymer PEDOT:PSS by electrochemical deposition, Na^+^ and K^+^ ISMs were deposited on top of the carbon fiber thread (CFT) electrodes by dip-coating the thread in the membrane cocktail. The reference electrode was obtained by coating the CFT electrode with Ag/AgCl ink and polyvinyl butyral (PVB). Both working and reference CFT electrodes were finally covered with the self-healing polymer, leading to an electrical healing ability with enhanced mechanical properties. The thread-based sensors for K^+^ (Figure 5A ii) and Na^+^ were tested separately in different buffer solutions containing the respective ion in the physiological concentration range (from 0.1 mM to 100 mM), showing sensitivities of 60.7 mV.log[Na^+^]^−^^1^ and 54.8 mV.log[K^+^]^−1^, respectively. The thread sensors were stable in a temperature range of 20–40 °C, under bending or crumpling, and showed good selectivity. Finally, the sensing threads were knitted onto a smart headband and connected to a flexible PCB, which acquired and transmitted data wirelessly to perform real-time sensing during physical activity, while the results were compared with the output of a standard electrochemical analyzer. The authors found out a similar response with both acquisition methods, thus indicating a high stability and reliability of the textile sweat sensor.

As an alternative to the standard potentiometric approach, OECT-based sensors can be employed in order to quantify the concentration of specific ions in liquid, including sweat. The groundbreaking work of Tarabella et al. [92] in 2012 presented for the first time a textile chemical sensor based on an OECT with a single PEDOT:PSS-coated cotton thread as the transistor channel. The thread was interfaced with a liquid electrolyte and a silver wire was employed as the gate electrode (Figure 5B i), the latter inducing a stable and reproducible modulation of the channel current. The simple and low-cost device was used to effectively sense the NaCl concentration in a liquid electrolyte, which could also be the body’s perspiration. The drain current could be modulated in the mA range by gate bias (Figure 5B.ii) and the relative current variation was directly tuned by the NaCl concentration in the liquid. Apart from that, it was not able to discriminate the presence of Na^+^ and Cl^−^ alone. Recently, in this vain, Coppedè et al. [93] proposed a similar textile-OECT device composed of a conductive channel made of a PEDOT:PSS-coated acrylic textile thread and a silver wire as gate (Figure 5C.i), able to selectively discriminate potassium and calcium concentration. In this case, the ion selectivity was achieved by exploiting ISM based on calcium ionophore (II) and potassium ionophore. In studying the two different tex-OECT devices’ performance (in terms of normalized response) separately, Na^+^, which is the most abundant electrolyte in human sweat, was used as an interferent to evaluate potassium and calcium sensors’ selectivity (Figure 5C ii). The K^+^ OECT sensor exhibited sensitivity values of 3.49 M^−1^ and 0.71 M^−1^ for K^+^ and Na^+^, respectively, while the Ca^2+^ OECT sensor showed sensitivities of 6.64 M^−1^ for Ca^2+^ and 0.81 M^−1^ for Na^+^. Despite these exciting results, the need to use an external, metallic gate electrode reduces the flexibility and portability of these devices.

A new and innovative approach has been reported by Gualandi et al. [94] to fabricate textile sensors inspired by OECT and thus overcome this problem. A novel composite material based on PEDOT:PSS and Ag/AgCl nanoparticles (NPs) embedded in the semiconducting polymer was synthesised, where the embedded NPs behave like nanogate electrodes. With a simple two-terminal configuration, the sensor exhibited the intrinsic amplification of a transistor during Cl^−^ detection. The textile sensor was made from a cotton yarn (Figure 5D i) coated with a PEDOT:PSS-based ink using a roll-to-roll-like technique. The NPs, consisting of an Ag core surrounded by a AgCl shell, were deposited on the polymer layer’s surface by a two-step electrodeposition process. The sensor behavior can be understood from the electronic coupling between the ionic charge and the electrochemically active nanoparticles. In fact, the spontaneous and reversible redox reaction between chloride and silver:

(1) $$Cl^{-}+Ag\;⇄AgCl+e^{-}$$

allowed one to monitor the chloride ion concentration in the sample solution with the expected Nernstian behavior (Figure 5D ii). The analytical signal that gave information about the Cl^−^ concentration was the current flowing in the functionalized conductive cotton thread, with a logarithmic response showing a sensitivity, expressed as normalized current, of (51 ± 9) 10^−3^ dec^−1^ in artificial sweat, short term response and selectivity to I^−^ and Br^−^ anions. Moreover, Kim et al. [26] reported another example of wearable OECT sensor based on PEDOT:PSS, exploiting a single-strand fiber-type for ion-concentration monitoring. The authors fabricated long, homogeneous and conductive microfibers by the wet spinning process from a sulphuric acid solution. The device consisted of a fiber-PEDOT:PSS channel and a Ag/AgCl wire as the gate electrode. The normalized drain current varied depending on NaCl concentration and the sensing performance was not related to the channel dimensions. Additionally, if attached onto human skin or embedded into clothes, the single-strand OECT maintained its mechanical and flexible properties. To achieve a more practical and easy-to-integrate sensor, the gate electrode was embedded directly onto the surface of the source connector in order to achieve a two-terminal single-strand wearable system. In this case, the device was composed by a PEDOT:PSS microfiber channel region with Ag wires at both ends sealed with silver epoxy and the non-active parts were passivated with spry-coating of poly-methyl methacrylate, while the gate electrode was realized on the source connection by chlorination onto the silver epoxy using Clorox 4% solution. This fabrication procedure led to a more convenient sensor configuration thanks to its source-gate hybrid electrode, which can impart an electrolyte concentration-driven potential to the active channel at zero gate bias in artificial or real human sweat. The calibration curve was acquired by linearly fitting the normalized channel current vs. the logarithm of the ion concentration and can be applied to perform real-sample detection by recording $\Delta I_{0}/I_{0}$ that is converted into the corresponding cation concentration value. Despite the microfiber OECT can perform repetitive measurement of cation concentrations, the value monitored in a real-human sample showed a systematic underestimation with a relatively large error of 10% if compared to mass spectrometry results, as only the monovalent cations can be detected.

### 4.3. Glucose and Lactate

Two of the most relevant analytes studied for personalized medicine are glucose and lactate because they are correlated to diseases or healthy states that are particularly widespread nowadays. The knowledge of glucose concentration is indispensable for hundreds of millions of people affected by diabetes to control the disease progress and correctly administer insulin. On the other hand, lactate is relevant for sport activities monitoring. It is well known that lactic acid is produced following an anaerobic metabolic pathway when low amounts of oxygen and/or nutrients reach cells. This condition is common during endurance sports activities, such as cycling, boxing, or running. If this anaerobic effort persists, it will induce an accumulation of lactate in the muscles, generating fatigue [14]. Another situation characterized by high lactate levels is observed when vessels are obstructed, for example, due to a combination of shear and friction or due to localized high pressure. If this obstruction is maintained for a long time, ischemia could happen, with the consequent death of cells and even necrosis of tissues.

Diabetic patients can self-test and monitor their blood glucose level by punching their skin and self-analyzing the results. The most popular commercial portable devices consist of disposable strips with a screen-printed electrochemical sensor and a pocket-size reader [95]. For lactate monitoring, commercial portable devices are also available. An example is the lactate SCOUT (Senslab) [96], which relies on an electrochemical method similar to the glucose one and based on blood samples. However, blood analysis has the main disadvantage of invasive and irregular frequency sampling. Even if this technology is widespread and approved for diabetic individuals, they would benefit from continuous non-invasive glucose monitoring in terms of therapy personalization. Similarly, in daily life, during sports activities or rehabilitation, continuous monitoring of lactate would provide important information to improve the training performance.

To reach this goal, several research groups have developed new sensors that exploit alternative body fluids instead of blood, with sweat being the most promising.

Diamond and co-workers developed SwEatch [97], which is the first example of a glucose sensor operating in sweat. Following this first experience, several other alternatives have been proposed [98]. Wang et al. reported tattoo-based electrochemical sensors for the detection of several biomarkers in sweat, including glucose [99] and lactate [100]. Similarly, Gao et al. [101] reported a wearable sweat multi-sensory system providing a comprehensive profile of sweat composition, enabling data cross-comparisons. Relevant results have been obtained by Rogers and co-workers, who developed stretchable sensors to continuously monitor lactate [102] and glucose [103] in sweat.

This huge work on new sweat sensors helped to demonstrate the existing correlation between glucose and lactate concentrations in sweat and blood. In the last decade, it has been demonstrated that the concentration of glucose in sweat is three orders of magnitude less than the concentration in blood and ranges between 10 µM and 1 mM [104,105].

On the opposite side, concentrations of lactate in sweat are even higher than the blood ones [106]. A typical reference level for a healthy state is 25 mM, however, this value strictly depends on several factors, such as body perspiration, age and gender [100]. Recent advances in wearable sensors have explored textile substrate to create functionalized fabric or fibers incorporated directly on clothes. Although colorimetric sensors have been developed [14,107,108,109], the direct conversion of chemical signals in electrical ones attracts more attention. Electrochemical biosensors based on glucose [17,110,111] and lactate oxidase [100,112] offer the highest reliability, due to the high selectivity associated with the use of an enzyme. In the specific case of glucose sensors, the main working principle is based on the immobilization of glucose oxidase (GOx) on the sensing element, which catalyzes glucose oxidation to gluconolactone:

(2) $$Glucose+O_{2}\overset{GOx}{\to }gluconolactone+H_{2}O_{2}$$

On the other hand, two different enzymes (lactate dehydrogenase (LDH) or lactate oxidase (LOx)) can be employed for lactate detection. In the presence of LDH, lactate is converted in pyruvate through its cofactor, nicotinamide adenine dinucleotide (NAD), with the following reaction:

(3) $$NAD^{+}+Lactate\;\overset{LDH}{\to }\;NADH+Pyruvate+\;H^{+}$$

Alternatively, LOx promotes lactate oxidation to pyruvate with concomitant release of H_2_O_2_ following the reactions:

(4) $$O_{2}+Lactate\;\overset{LOx}{\to }\;Pyruvate+\;H_{2}O_{2}$$

Once the enzymes have catalyzed glucose or lactate oxidations, the produced electrons can be transferred to the electrode surface by using either the natural products of the reactions or by taking advantage of a redox mediator that is immobilized on the electrode surface. In some cases, direct electrical contact between the redox centers of the enzyme and the electrode can be obtained.

CPs are functional materials that play a key role in the development of OECT-based biosensors, which are able to directly amplify the electrochemical signals generated by working electrodes modified with LOx and GOx.

The gate electrode is typically functionalized with GOx or LOx to oxidize glucose or lactate, respectively [113], to generate an electrical signal following the same pathways of amperometric sensors, with the major advantage that no reference electrode is required when using a transistor configuration. The enzyme catalyzes the oxidation event that generates a change in the effective gate voltage and affects the channel current [114,115,116].

**Figure 6:**
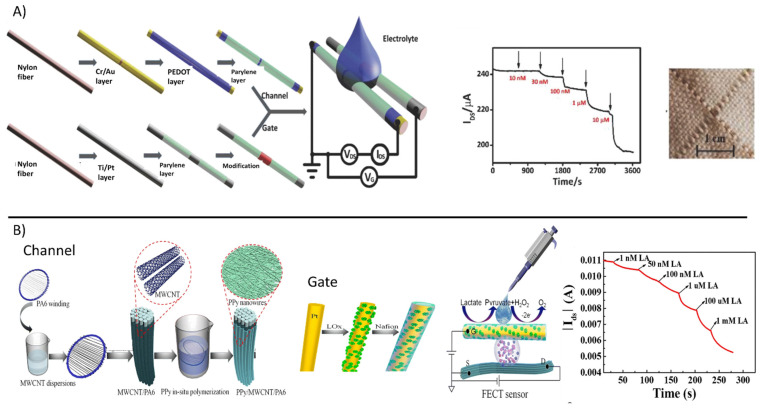
(**A**) Fabrication process of the nylon fiber OECT for glucose sensing. The channel is obtained by the deposition of Cr/Au on a nylon fiber, followed by a PEDOT layer and parylene coating. The gate is obtained by the deposition of Ti/Pt and parylene coating, then modified with chitosan and GOx. Bottom right, graph of the glucose sensor response. Bottom left, photo of the fiber OECT integrated in fabrics. (reproduced from [117] with permission from Elsevier). (**B**) Fabrication process of the fiber based OECT for lactate sensing. From left to right, PA filament is immersed in MWCNT dispersion, (circle zoom shows MWCNT/PA6 filament), then PPy is polymerized in-situ, obtaining a PPy/MWCNT/PA6 fiber channel (circle zoom shows PPy nanowires). The gate electrode is obtained from a Pt wire, coated with LOx and protected with Nafion membrane. Scheme of the fiber based OECT sensor for lactate sensing. Sensors response to lactate additions. Reprinted by permission from [119], Copyright (2021) Springer Nature.

Wang et al. [117] proposed a fiber-based OECT made of in situ polymerized polypyrrole (PPy) nanowires and reduced graphene oxide (rGO) (Figure 6A). The fiber gate electrode was further functionalized with GOx and Nafion, the large surface area of PPy nanowires improving GOx loading and leading to fast electron transfer and high sensitivity. Glucose, which was dissolved in the LiClO_4_ gel electrolyte in contact with the gate and channel, was oxidized at the GOx-modified gate electrode and, concomitantly, the H_2_O_2_ released as a side-product reacted with the strong oxidizing agent LiClO_4_, thus enhancing the conductivity of the fiber-based OECT channel. As a consequence, glucose was detected as an increase in the drain current. The intrinsic amplification of the fiber-based OECT configuration allowed it to reach an outstanding sensitivity of up to 0.773 decade^−1^ (expressed as a normalized response) and a limit of detection (LOD) down to the nanomolar range.

Yang et al. [118] described a fiber-based OECT sensor for glucose based on conductive nylon fibers. The channel was fabricated from a nylon fiber. Cr/Au was firstly deposited, followed by PEDOT:PSS dip-coating. The best performing gate electrode was obtained by chemically modifying a Ti/Pt nylon fiber with GOx, chitosan, and graphene flakes. Gate and channel fibers were woven with cotton yarns resulting in stretchable fabric biosensors. The fiber-based OECT biosensor exhibited relevant performance with a low LOD of 100 nM and a stable linear response up to 300 μM, being suitable for sweat analysis application. Similarly, a gate electrode functionalized with LOx was employed for the lactate sensors. Zhang et al. [119] reported a novel approach to fabricate a fiber-based OECT biosensor (Figure 6B). A nylon (PA6) fiber was first dip-coated with multi-walled carbon nanotubes (MWCNTs) and then in-situ polymerized PPy, leading to a composite material, e.g., PPy/MWCNTs, with a large surface area that enhanced stability and the on/off ratio with respect to the simple PPy-coated fiber. The PPy-MWCNTs fiber was used as the channel of OECT, while either PPy-MWCNTs fiber and a Pt wire coated with LOx immobilized in a Nafion matrix were tested as gate electrodes. The OECT biosensor was biased with a gate voltage equal to 1.0 V to stimulate the oxidation of hydrogen peroxide produced during lactate oxidation. Similar to previous reports, the electrons released at the gate electrode generated a Faradaic current and changed the effective gate voltage, which directly affected the channel conductivity. The Pt/Nafion/LOx fiber gate electrode showed the best performance when compared to the simple PPy-based counterpart, with a wide linear response range of 1 nM–1 mM suitable for sweat analysis.

### 4.4. Multi-Sensing Platforms

**Figure 7:**
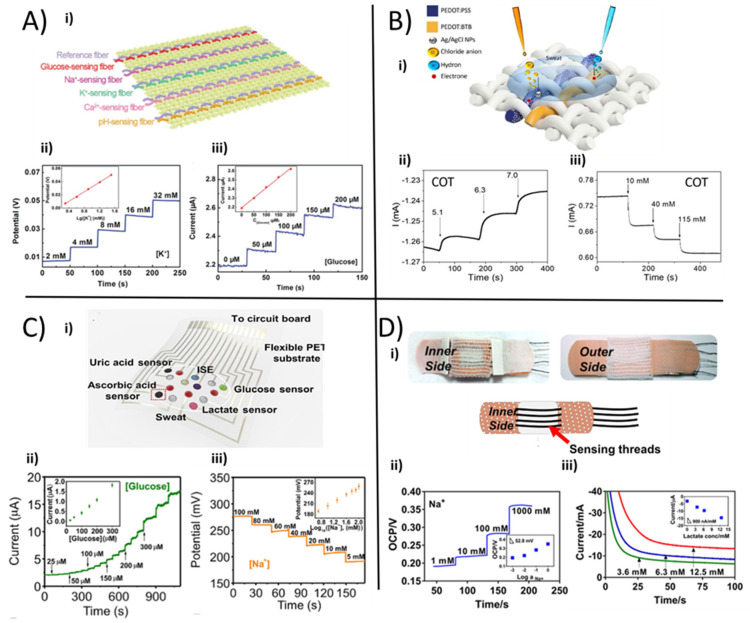
(**A**) i. Schematic illustration for the fabrication of the electrochemical fabric by weaving sensing fibers; ii. the open-circuit-potential (OCP) responses of the K^+^ sensor to analyte additions, iii. the chronoamperometric response of the glucose-sensing fiber in PBS (reproduced from [36], Copyright (2021) Wiley). (**B**) i. Illustration of the textile multi-thread sensor platform. Current vs. time plot for cotton-based (ii) chloride and (iii) pH sensor in universal buffer solution [83]. (**C**) i. Photograph of the wearable sweat analysis patch; (ii) chronoamperometry response of silkNCT-based glucose sensor; (iii) OCP response of sodium sensor. The inset graphs are the calibration plots of the sensors [120]. (**D**) i. Photograph of the patch sensor prototype; ii. potentiometric response to sodium additions and iii. Chronoamperometric signal of lactate using filter paper soaked in proper analyte solutions [121].

The ability to measure and analyze in real-time critical health-related parameters in a non-invasive way opens up new alternatives and new scenarios for obtaining flexible, selective, and highly sensitive multi-sensor platforms. Unconventional substrates such as fabric and fibers have been used in numerous studies to manufacture unique, flexible and wearable biosensors. As follows, recent examples of textile platforms are presented that combine the possibility to detect various biomarkers and analytes in human sweat with the well-known comfortability of textiles. Exploiting the multiple advantages of CPs, coupled with carbon-based material and enzymes, it is possible to develop textile platforms that are able to monitor numerous parameters such as electrolytes, pH, and metabolites to perform a physiological diagnosis of cystic fibrosis, hydration status, bone mineral loss, or physical stress. Incorporating additional sensors in the same textile sensing system makes it possible to perform simultaneous analytes detection from a single sample. In 2018, Wang and co-workers [36] reported one of the first examples of a multi-analyte platform, exploiting a novel approach to realize an electrochemical fabric from a single sensing fiber. Specific biologically relevant compounds, such as glucose, pH, Na^+^, Ca^2+^, and K^+^, were efficiently detected directly from human perspiration exploiting a set of textile fiber sensing units, which were stitched together, as shown in Figure 7A i. The sensors were realized in a coaxial manner, in which the internal core, made of CNT fibers, was coated with the sensing layer. In particular, PANI and PEDOT:PSS were used in order to exploit their flexibility, good conductivity and sensing capability. The electroplating technique was used to realize the pH-sensing fiber depositing PANI onto the external surface of CNTs. The glucose-sensitive thread was made by a drop-casted layer of chitosan/SWCNTs/GOx directly deposited onto the CNTs and an electrochemically deposited layer of Prussian Blue (PB) as a redox mediator to provide better sensitivity. The ion-sensing fibers were realized exploiting a galvanostatically polymerised film of PEDOT:PSS as an ion-to-electron transducer, on which an ion-selective ionophore was drop-casted as a specific ion detector. Sodium, calcium, and potassium ion-sensing fibers were integrated into the multiplex-sensing electrochemical fabric to monitor the liquid’s electrolyte concentration.

Fiber reference electrodes made of CNT fibers, which were coated with an Ag layer and PVB following a chlorination step, were twisted together with ions and pH sensing working electrodes, so as to allow the potentiometric set-ups to be weaved into a fabric. When tested in the respective electrolyte solutions, these sensors showed a linear and reproducible relationship in the physiological human range with sensitivities of 45.8 mV.dec^−1^, 35.9 mV.dec^−1^, and 52.3 mV.dec^−1^ for Na^+^, K^+^, and Ca^2+^ respectively.

Furthermore, the amperometric glucose-sensing fiber was characterized in phosphate buffer solution (PBS) and exhibited a linear response, including typical sweat levels with a sensitivity of 2.15 nA.µM^−1^. Figure 7A ii–iii report an example of the fiber sensor response.

To show the actual application, the authors performed an on-site analysis that integrated the sensory tissue into the clothing to test and directly analyze human perspiration.

Recently, Possanzini et al. [83] reported a fast, cost-effective and innovative strategy to fabricate a textile multi-sensor platform that merged a versatile and referenceless geometry structure with the robustness of a potentiometric-like transduction mechanism. The multi-thread sensing platform sketched in Figure 7B i could stably measure pH (Figure 7B ii) and Cl^−^ concentration (Figure 7B iii) simultaneously and selectively. Different commercial threads, e.g., cotton, polyester and silk, were tested after PEDOT:PSS coating using a roll-to-roll method. Afterwards, a proper functionalization with either Ag/AgCl NPs [81] or PEDOT:BTB [26] was carried out to obtain Cl^−^ and pH selective sensing functionality, respectively. The simultaneous in-vitro characterization in artificial human sweat of both thread-sensors gave important results in terms of reliability, selectivity, and sensitivity, expressed as normalized current, with a repeatable value of (75 ± 2) 10^−3^ dec^−1^ and (12 ± 2) 10^−3^ pH^−1^ for Cl^−^ and pH, respectively.

He et al. [120] provided another important example of a soft and flexible silk fabric-derived intrinsically nitrogen (N)-doped carbon textile (silkNCT), able to detect and measure the level of six different health-related biomarkers in human fluids. Silk fabrics were based on homogeneous and mechanically robust silk fibers that can be transformed into flexible, highly conductive, and nitrogen-doped carbon fabrics by proper heat treatment. The silkNCT textile, opportunely shaped in a circle, was used to realize the sensing substrate to achieve a multi sensor array to simultaneously and selectively detect Na^+^ and K^+^, lactate, glucose, uric acid (UA) and ascorbic acid (AA). For the biomolecules, a conventional amperometric approach was employed, comprising clipped silkNCT pieces as the working electrodes, Ag/AgCl ink-modified conductive tape as a reference and silkNCT part as a counter electrode. For ions detection, a two-electrode system was adopted. Enzyme-based amperometric sensors were employed for glucose and lactate detection in human sweat, where the silkNCT fabric was coated with Pt NPs by electrochemical deposition and then functionalized with GOx or LOx by drop-casting. The ion sensors were fabricated exploiting the typical ISM receipts. The ISMs were drop-casted onto the corresponding electrode of silkNCT, which was previously coated by galvanostatic electrochemical polymerization with PEDOT:PSS, with the function of minimizing the potential drift the during experiments. Figure 7C i reports the scheme of the wearable analysis patch, and Figure 7C ii–iii shows the responses of sodium and glucose sensors, respectively.

When tested in PBS (pH 7.0), the lactate and glucose flexible and textile patches presented a sensitivity of 174 nA.µM^−1^ and 6.3 nA.µM^−1^, respectively, while the electrolytes sensors had both sub-Nerstian behavior with a linear trend in the physiologically relevant human range. UA and AA sensors reported a sensitivity of 196.6 nA.µM^−1^ and 22.7 nA.µM^−1^, respectively. As a proof of concept for a real-file application, the wearable patch was embedded in a wristband to record real-time sweat analysis during sports exercise with wireless data communication with a smartphone.

Additionally, Terse-Thakoor et al. [121] has reported a thread-based multiplex sensor patch for real-time sweat monitoring (Figure 7D i). In particular, such kinds of fibrous textile threads could be conveniently integrated in fabric and adhesive patches to perform on-body and real-time tracking of essential biomarkers present in human sweat, such as sodium and ammonium ions, lactate, and pH. The flexible threads were coated with carbon ink and Ag/AgCl inks to realize working and reference electrodes, respectively. NH_4_^+^ and Na^+^ sensors were fabricated by dip-coating a carbon/polyethylene (PE) yarn in ion-selective membrane cocktails and made use of a solid-state reference thread electrode based on a PVB-coated Ag/AgCl/PE thread. The pH sensing thread was realized by potentiostatic deposition of PANI on the surface of a carbon-coated stainless steel (SS) thread. Finally, the lactate sensor was realized with a PB-coated PE thread, which was successively modified with chitosan and LOx by drop-casting. Figure 7D ii–iii reports sodium and lactate sensor responses after a separated evaluation in standardized solutions. The Na^+^/C/PE, NH_4_^+^/C/PE, and PANI/C/SS threads showed Nerstian behavior with sensitivities of 52.8, 60.6, and 62.3 mV.decade^−1^, respectively. In the physiologically relevant range, the chronoamperometric response of LOx/PB/PE sensor reached a sensitivity of 900 nA.mM^−1^.

The sensors also showed good selectivity, reproducibility, and low hysteresis. The sensor patch was coupled with an electronic board for a wireless data acquisition and data processing to achieve a wearable and real-life sweat monitor platform, which was then tested during on-body trials.

### 4.5. Other Analytes

In addition to the predominant and most common analytes, sweat contains a variety of trace biomarkers such as metabolites and organic compounds whose detection, despite being relevant to wearable technologies for health care monitoring, has been scarcely explored. The reason might be sought either in their low/ultra-low concentration in sweat, or in the complex realisation of efficient transduction strategies using textile substrates.

Among them, adrenaline is an amino acid-derived hormone, which is produced by the adrenal glands in the presence of alert or stress situations and under strong physical conditions. The quantification of adrenaline concentration in sweat is under study [122]. Two examples of textile chemical sensors for adrenaline determination have been reported to date, where its oxidation to adrenochrome is exploited for quantitative detection. Coppedè et al. [123] developed a wearable OECT sensor where a metal yarn and a PEDOT:PSS-coated cotton fiber were the gate electrode and the transistor channel, respectively. By endowing two distinct channels with Pt or Ag wire gate electrodes, the authors were able to play with the faradaic and non-faradaic operation regimes of the OECTs and demonstrated the possibility to independently detect adrenaline (10^−3^–10^−6^ M concentration range) and NaCl in human sweat with the two sensors sewn close to each other on the same fabric patch. In fact, adrenaline electrooxidation can only take place at the Pt wire gate electrode, while the Ag-OECT is sensitive to ions. In order to design a fully-textile platform for sweat sensing, Gualandi et al. [20] exploited the electrocatalytic properties of PEDOT:PSS to design an all-PEDOT:PSS OECT-based sensor on a woven cotton fabric. Here, the ability of the organic gate electrode to catalyse the electrooxidation of adrenaline, dopamine (DA) and AA and to produce concentration-dependent variations in the transistor output current was demonstrated in artificial sweat. Importantly, the direct screen-printing of PEDOT:PSS gate and channel allows the seamless integration of the sensor into the textile substrate and detection limits of 10^−8^ mol were reached for the three biomarkers by the textile sensor in the optimised geometry.

Like adrenaline, DA quantification in human sweat is under study [72], while AA concentration was reported to vary in the range 1.4–62.5 μM [122]. A fiber-based organic electrochemical transistor (FECT) made of nylon fibers coated by poly(vinyl alcohol-co-ethylene) nanofibers and electrodeposited PPy nanofiber network was also reported for DA sensing [124]. The woven FECT was able to detect DA in the range 1 nM–1 μM exploiting its oxidation at the PPy-based gate electrode and showed very good specificity in the presence of NaCl, UA, AA and glucose, however, the origin of the selective response was not analyzed. Another attractive biomarker for sweat analysis is creatinine, a metabolic product of medical relevance for the diagnosis of renal insufficiency whose concentration in human sweat typically ranges from 0.9 to 115 μM [72]. Recently, a textile amperometric sensor for environmental heat-stress sweat creatinine monitoring was reported based on nylon coated by poly(vinyl alcohol) (PVA)-Cu^2+^-PEDOT:PSS and Cu_2_O NPs [125]. The proposed sensing mechanism relied on the diffusion of creatinine across the cross-linked conductive system, followed by catalytic binding with Cu^+^ ions attached to the thiophene rings of PEDOT, leading to a high selectivity towards creatinine from 0.4 to 960 μM in human sweat sample.

The typical concentration of the amino acid L-tyrosine in human sweat is around 1.0 mM [72] and unbalanced levels may be a fingerprint of severe metabolic disorders. A proof-of-concept study has been reported on an OECT-based textile biosensor for tyrosine obtained by the non-covalent immobilisation of the enzyme laccase at a PEDOT:PSS coated cotton fiber used in place of the transistor channel, while a Pt wire was the gate electrode [126]. Thanks to the three-dimensional fiber network and the excess of charged PSS^−^ units promoting electrostatic interactions, the authors claimed that the resulting orientation of laccase molecules was favourable to realise the effective direct electron transfer. The performance of the wearable OECT biosensor was tested in citrate buffer, where a 10^−8^ M tyrosine addition was detected.

## 5. Challenges

CPs could play a key role in the development of wearable chemical sensors because they merge electrical conductivity with the mechanical features necessary to be embedded in textiles, thus achieving a wear-and-forget functionality. Despite these peculiar features, further efforts should be devoted to the research on CP-based textiles to meet the standards required for commercialization and, thus, to have an impact on real-life.

The main challenges concerning material development deals with maintaining their functionalities during their real use in a relevant environment. The sensor response should be unchanged after the deformations occurring during the wearing and the electrical features of each element should be preserved after repeating bending and stretching. Moreover, textile chemical sensors should endure normal washing cycles that must be performed on clothes in real-life. The design of devices and materials will significantly contribute to reach this goal. For example, Maity et al. [127] described a textile ammonia chemosensor based on a composite material composed of PANI and MWCNTs that allows the device to maintain the same response also after deformation. On the other hand, the literature takes advantage of the strengthened binding between textiles and functional materials to produce washable e-textiles based on innovative active materials. For example, Wang’s group reported on a textiles’ pre-treatment to obtain a hydrophobic substrate that is water-resistant [76]. Yun et al. described NO_2_ yarn sensors, which maintain good sensing performance after 10 washing cycles [128]. Before depositing the conductive materials, yarns were covered with bovine serum albumin. Additionally, the covalent immobilization of dyes in cellulose-based textiles can be employed to reach remarkable stability to washing cycles [129].

Although the main challenges are focused on the sensor stability under mechanical stress or washing, fouling or hysteresis effects affecting the sensing process could be detrimental to the device response as well. Moreover, the design of transducing materials should be carried out bearing in mind the need to develop ready-to-use, maintenance-free and user-friendly devices suitable for non-expert operators. In fact, calibration procedures should be minimized or avoided, and the sensor response should have full reversibility with no cleaning processes required. Thereby, the stability during sensing should also be considered in real applications.

Last but not least, textiles can also act as active elements in sensing processes and play a more important role than the simple substrate where the device is deposited. The fabric’s ability to absorb liquids can be exploited to generate a capillarity gradient, so as to induce a sweat flow that starts from the skin surface and reaches a storage system passing close to the transducer of the device. Thereby, the sample is continuously renewed close to the sensing element avoiding the stagnation that could invalidate the measurements. He et al. [130] have developed a self-pumping apparatus based on janus textile that is employed as a sampling system in sensors for the detection of glucose, lactate, K^+^, and Na^+^.

## 6. Conclusions

Wearable textile sensors are a promising new research topic due to the potential application in the fields of medicine, sports activities, and occupational and health safety. The growth of technology readiness level is limited by several bottlenecks that can be broadly ascribed to materials, power supply, data acquisition and processing, communication, and analytical requirements. CPs are a class of materials that could help to overcome the main limitation concerning fabrication, because they exhibit the conformability of polymers and electrical conductivity that lies in the range of semiconductors. On one hand, CPs are not very exploited in the field of amperometric transduction, where the few examples concern the development of multisensory arrays. On the other hand, the design of potentiometric textile sensors profits from both the CPs’ ability as a solid contact transducer and the peculiar chemistry of PANI in pH sensing. Such devices take advantage of a mature technology in electrochemical sensing, wherein CPs operate in the same ways, to overcome the limitations represented by the stiffness and rigidity of other sensing materials. However, CPs also offer fascinating solutions to other aforementioned bottlenecks. They are the functional materials that allow the production of transistor-based sensors, whose output is pre-amplified with a consequent simplification of the signal acquisition. Moreover, the OECT architecture can be easily embedded in a textile due to the lack of a reference electrode. Finally, the synthesis of new CP-based materials is an intriguing way to face the challenges concerning the development of textile sensors. The combination of CPs and carbon nanomaterials allows one to reach a stability of electrical performance under mechanical stress. On the other hand, the design of novel sensing materials enables the production of transistor-inspired devices, wherein an electrochemical gating effect controls the CP electrical conductivity as a function of the analyte concentration. These sensors simply operate with a single element, which might be a yarn, with a consequent simplification of their architecture and fabrication process.

## Figures and Tables

**Table 1:** The typical ions concentration in human sweat.

Metabolites	Concentration Range (mM) in Sweat	Ref.
Ca^2+^	0.07–12	[64]
Potassium	4–24	[65]
Chloride	10–100	[66]
Sodium	10–100	[67]
Ammonium	0.5–8	[67]
Lactate	5–60	[68]
Urea	14.2–30.2	[69]
Glucose	0.01–1	[70]
Ethanol	2.25–22.5	[71]
Pyruvic acid	0.06–1.6	[72]
Amino acids *	303	[72]
Ascorbic Acid	0.01–0.03	[73]

* weighted average value.
